# BNIP3 mediates the different adaptive responses of fibroblast-like synovial cells to hypoxia in patients with osteoarthritis and rheumatoid arthritis

**DOI:** 10.1186/s10020-022-00490-9

**Published:** 2022-06-11

**Authors:** Ran Deng, Yan Wang, Yanhong Bu, Hong Wu

**Affiliations:** 1 Key Laboratory of Xin’an Medicine, Ministry of Education, Hefei, 230012 China; 2 Anhui University of Chinese Medicine, Qian Jiang Road 1, Hefei, 230012 China; 3 Anhui Province Key Laboratory of Research &, Development of Chinese Medicine, Hefei, 230012 China; 4 College of Pharmacy, Anhui University of Chinese Medicine, Hefei, 230012 China

**Keywords:** BNIP3, Mitophagy, Apoptosis, Fibroblast-like synovial cells, Rheumatoid arthritis

## Abstract

**Background:**

Hypoxia is one of the important characteristics of synovial microenvironment in rheumatoid arthritis (RA), and plays an important role in synovial hyperplasia. In terms of cell survival, fibroblast-like synovial cells (FLSs) are relatively affected by hypoxia. In contrast, fibroblast-like synovial cells from patients with RA (RA-FLSs) are particularly resistant to hypoxia-induced cell death. The purpose of this study was to evaluate whether fibroblast-like synovial cells in patients with osteoarthritis (OA-FLSs) and RA-FLSs have the same adaptation to hypoxia.

**Methods:**

CCK-8, flow cytometry and BrdU were used to detect the proliferation of OA-FLSs and RA-FLSs under different oxygen concentrations. Apoptosis was detected by AV/PI, TUNEL and Western blot, mitophagy was observed by electron microscope, laser confocal microscope and Western blot, the state of mitochondria was detected by ROS and mitochondrial membrane potential by flow cytometry, BNIP3 and HIF-1α were detected by Western blot and RT-qPCR. The silencing of BNIP3 was achieved by stealth RNA system technology.

**Results:**

After hypoxia, the survival rate of OA-FLSs decreased, while the proliferation activity of RA-FLSs further increased. Hypoxia induced an increase in apoptosis and inhibition of mitophagy in OA-FLSs, but not in RA-FLSs. Hypoxia led to a more lasting adaptive response. RA-FLSs displayed a more significant increase in the expression of genes transcriptionally regulated by HIF-1α. Interestingly, they showed higher BNIP3 expression than OA-FLSs, and showed stronger mitophagy and proliferation activities. BNIP3 siRNA experiment confirmed the potential role of BNIP3 in the survival of RA-FLSs. Inhibition of BNIP3 resulted in the decrease of cell proliferation, mitophagy and the increase of apoptosis.

**Conclusion:**

In summary, RA-FLSs maintained intracellular redox balance through mitophagy to promote cell survival under hypoxia. The mitophagy of OA-FLSs was too little to maintain the redox balance of mitochondria, resulting in apoptosis. The difference of mitophagy between OA-FLSs and RA-FLSs under hypoxia is mediated by the level of BNIP3 expression.

## Introduction

Physiological hypoxia refers to the environment with relatively low oxygen concentration in normal embryonic development and brain tissue, which is essential for the exercise of the normal functions (Taylor and Colgan 2017). While the disturbance of normal oxygen supply leads to a series of diseases (Hao et al. 2022; Veldhuizen et al. 2021). In the process of excessive synovium hyperplasia, severe hypoxia of local tissue due to excessive proliferation is one of the important characteristics of rheumatoid arthritis (RA) joint microenvironment (Jiang et al. 2021; Guo and Chen 2020). Different types of cells respond differently to hypoxia. Hypoxia induces adaptive response to maintain the homeostasis of intracellular environment and promote cell survival (Kong et al. 2022; Qureshi-Baig et al. 2020). For others, hypoxia induces cell death by activating apoptosis (Ren et al. 2021; Mueller-Buehl et al. 2021). The research on the changes of metabolic function caused by hypoxia and its mechanism will help to understand the pathogenesis of hypoxic diseases.

Hypoxia-inducible factor 1 (HIF-1) is a heterodimeric transcription factor that regulates the body’s response under hypoxia, and its transcription activity is determined by the HIF-1α (Ikeda et al. 2021). Under hypoxia, HIF-1α is stably expressed to activate the expression of target genes (Huang et al. 2021). Studies have confirmed that HIF-1α activated mitophagy by up-regulating its target gene Bcl-2 and adenovirus E1B 19 kDa interacting protein 3 (BNIP3) (Fu et al. 2020). The role of BNIP3 in cell death is controversial. BNIP3 induces apoptosis and mitophagy, or even stimulates cell proliferation (Lin et al. 2021; Gorbunova et al. 2020). Fundamentally, cell type and environment determine the interdependence between autophagy and apoptosis. Studies have confirmed that mitophagy is an important hypoxic adaptive response, which selectively degrades damaged mitochondria and maintains redox balance to prevent cell death. Different types of cells may have different adaptation to hypoxia by regulating the expression of BNIP3 (Filippi et al. 2018).

As the most common inflammatory arthritis, the pathological feature of RA is that the pannus composed of synovial cell layer and angiogenesis invades into the joint cavity or cartilage to cause joint damage. Synovitis was previously thought to be driven immune inflammation mediated by T lymphocytes (Weyand et al. 2014). Then it was found that there was joint bone destruction mediated by rheumatoid arthritis fibroblast-like synovial cells (RA-FLSs) (Cai et al. 2022; Nygaard and Firestein 2020). RA-FLSs themselves have a series of transformation characteristics, with blocked growth regulatory mechanisms and reduced apoptosis, which lead to “tumor-like” growth and hyperproliferation (Wang et al. 2020a, b). The characteristics of abnormal proliferation of RA-FLSs is consistent with the pathological features of abnormal synovial hyperplasia. Their survival depends not only on the stimulation of growth factors in the synovial microenvironment, but on hypoxia (Lee et al. 2018; Yang et al. 2022). Synovial hyperplasia is a persistent marker of RA progression. The excessive proliferation of RA-FLSs plays a crucial role in the progression of RA. It was reported that BNIP3 was widely expressed in patients with RA, and its expression was positively correlated with the severity of the disease (Kammouni et al. 2007).

We established an in vitro hypoxia model to explore the different responses of fibroblast-like synovial cells in patients with osteoarthritis (OA-FLSs) and RA-FLSs to hypoxia in terms of proliferation, apoptosis and mitophagy, and to explore whether BNIP3 mediated the different adaptive responses of OA-FLSs and RA-FLSs.

## Materials and methods

### Cell culture and hypoxic treatment

RA-FLSs are a cell line isolated from synovium of patients with RA, and it was purchased from BeNa Culture Collection (Suzhou, China). RA-FLSs were inoculated in DMEM medium (Hyclone, New Zealand) containing 10% (v/v) fetal bovine serum (FBS, Biological Industries, Israel), 1% (v/v) 100 U/mL penicillin and 100 LG/mL streptomycin (Beyotime, Shanghai, China). OA-FLSs were isolated from the synovium of osteoarthritis patients and purchased from Bena Culture Collection (Suzhou, China). Cells were cultured as normoxia group at 21% O_2_, 74% N_2_, and 5% CO_2_. Cells was exposed to 2% O_2_, 93% N_2_, and 5% CO_2_ in a hypoxia incubator chamber (AIPUINS, USA) for 24 h as an hypoxia group (Touhami et al. 2022).

### Cell proliferation assay

As previously described, cell viability was determined by cell counting kit-8 (CCK-8) assay. Briefly, OA-FLSs or RA-FLSs (5 × 10^4^ cells/mL) were inoculated in 96 well plates, and cells were cultured in an incubator at 37 °C and 5% CO_2_. The hypoxia condition of 2% O_2_ was established by placing cells in a hypoxia incubator chamber. After the culture, 10 μL of CCK-8 (Solarbio, Beijing, China) was added, cultured in a 5% CO_2_ at 37 °C for 1 h, and the optical density (OD) was measured at 450 nm.

### Cell cycle analysis

Cells were cultured in the corresponding oxygen concentration for 24 h, and cell precipitation was collected after trypsin digestion. After cells were washed with PBS, 75% cold ethanol was added and fixed overnight at 4 °C. Cells were centrifuged again, the precipitates were washed with PBS for three times and resuspended at 200 μL in PBS. According to the instructions provided by the manufacturer, 2 μL RNase A with a concentration of 10 mg/mL was added to the cell suspension for digestion for 30 min, and then 300 μL of propidium iodide (PI) at a concentration of 50 μg/mL incubated in dark for 30 min (Li et al. 2022). The results of cell cycle were detected by flow cytometry, and results were analyzed quantitatively with FlowJo 7.6 software.

### BrdU assay

OA-FLSs/RA-FLSs (5 × 10^4^/mL) were cultured in a 24-well plate. After 24 h of culture, 500 μL of BrdU (10 μM) was added to each well and then transferred to the corresponding oxygen concentration incubator for incubation for 1.5 h (Liu et al. 2021). After BrdU labeled cells were completed, the original culture medium was sucked and discarded, and 500 μL 4% paraformaldehyde was added at room temperature for 15 min, and then infiltrated with 0.5% Triton X-100 for about 20 min. Finally, Hoechst staining was used for 15 min to label the nucleus. Subsequently, samples were observed under a fluorescence microscope (Olympus, Japan).

### Annexin V-FITC/PI assay

Annexin V-FITC/PI double staining detection kit was used to evaluate the induction of apoptosis. OA-FLSs or RA-FLSs were inoculated in 35 mm dishes. Resuspended OA-FLSs or RA-FLSs in 400 μL of 1× Binding buffer solution and adjusted the cell density to 1 × 10^6^/mL. Added 5 μL of AV-FITC to the cell suspension and incubated for 10 min at room temperature in the dark, then added 10 μL of PI and incubated for 5 min at room temperature in the dark (Bestbio, Shanghai, China), and samples were read by flow cytometry (Zhou et al. 2021).

### TUNEL assay

TUNEL cell apoptosis detection kit was used to detect the breakage of nuclear DNA in the late stage of apoptosis. TUNEL positive cells were detected according to the manufacturer’s instructions (Servicebio, Wuhan, China). Added 500 μL of 4% paraformaldehyde to fix for 20 min, and after washing, added 500 μL of 0.3% Triton-X-100 to each well and incubated for 15 min at room temperature; aspirated and discarded the permeabilizer, added 100 μL of 1× DNase I Buffer and incubated for 5 min at room temperature. Added 100 μL of working solution containing DNase I (20 U/mL) and incubated for 10 min at room temperature. After washing, added 50 μL of quilibration Buffer to each sample to completely cover the slide, and incubated at room temperature for 10 min. Prepared enough TdT incubation buffer for all experiments according to Recombinant TdT enzyme, FITC-12-dUTP Labeling Mix and Equilibration Buffer at the ratio of 1 μL:5 μL:50 μL. Added 56 μL of dT incubation buffer to the samples, incubated at 37 °C for 1 h in the dark, washed and then added 500 μL of 10 μg/mL DAPI solution to room temperature for 8 min to stain the cells (El-Habta et al. 2021).

### Mitochondrial membrane potential (MMP) polychromatic assay

MMP was measured with cationic JC-1 (5′,6,6′-tetrachloro-1,1′,3,3′-tetraethyl benzimidazolyl carbocyanine iodine) dye. Intact polarized mitochondrial membrane showed red signal (JC-1 aggregate), while green signal (JC-1 monomer) was depolarized mitochondrial membrane. After OA-FLSs or RA-FLSs were placed in a 35 mm Petri dish for corresponding treatment, the cell precipitates were collected. Briefly, added 1 mL of JC-1 staining working solution, incubated at 37 °C for 20 min in a cell incubator, removed the supernatant, and washed twice with JC-1 staining buffer (1×). Added 1 mL of cell culture medium to resuspend the cells (Solarbio, Beijing, China) (Li et al. 2019), and then the samples were examined by flow cytometry. Results of flow cytometry were analyzed using the FlowJo software 7.6.

### Reactive oxygen species (ROS) assay

The evaluation of ROS production in OA-FLSs or RA-FLSs was labeled with DCFH-DA probe (Beyotime, Shanghai, China), and the cells were added at a concentration of 10 μM/mL DCFH-DA medium and incubated at 37 °C for about 20 min. Then, the cells were washed three times with serum-free medium to remove the remaining probes. Cell precipitates were collected and samples were analyzed by flow cytometry.

### Transmission electron microscopy

For transmission electron microscopy analysis, the cell precipitates of OA-FLSs/RA-FLSs were rapidly fixed in 2.5% glutaraldehyde, then fixed in 1% osmium tetroxide, dehydrated with graded ethanol and embedded in hard resin. Next, they were cut into ultra-thin slices (80 nm) using an ultra micro slicer (Leica, Germany). Then, before TEM observation, the sections were stained with uranyl acetate and lead citrate, and the occurrence of mitophagy was determined according to previous reports (Fu et al. 2020).

### Laser-scanning confocal microscopy

OA-FLSs/RA-FLSs were transfected with green fluorescence protein microtubule-associated protein 1 light chain 3B (EGFP-LC3B) plasmid (genomeditech, Shanghai, China) for autophagy. Mitotracker red (a special mitochondrial marker, c1035, beyotime, Shanghai, China) with a concentration of 100 nM was incubated at 37 °C for 30 min, and then treated with DAPI for 5 min (10 μg/mL, 5 min, 37 °C, Biosharp, China) (Li et al. 2019). RA-FLSs were transfected with BNIP3-lowexpression plasmid or vector under normoxia for 24 h before the staining with MitoTracker Red and DAPI. Added 1 mL of 4% paraformaldehyde to FLSs after hypoxia treatment, fixed the cells at room temperature and let them stand for 30 min. After washing the cells with PBS, add 1 mL of 0.1% Triton-x 100 to permeabilize the cells at room temperature for 15 min, washed them with PBS for three times, then made cell climbing slices. Cells were observed with confocal laser scanning microscope (ZEISS, Oberkochen, Germany).

### Western blot

OA-FLSs/RA-FLSs were promptly processed to avoid culture reoxygenation. First, the cells were washed three times under PBS at room temperature and then lysed in Ripa buffer containing 1 mM phenylmethylsulfonyl fluoride. Subsequently, the cells were centrifuged at 4 °C for about 30 min to obtain the supernatant. The extracted total protein was quantified by BCA protein quantitative kit (Beyotime, shanghai, China), and then the sample was loaded according to the quantitative results to ensure that the total protein in each lane was 50 µg. The target protein was separated by 12% SDS–polyacrylamide gel. Western blot was performed as previously described (Deng et al. 2021). The main antibodies used for immunoblotting are as follows: Bax (1:1000, Proteintech), Bcl-XL (1:1000, Proteintech), Beclin-1 (1:1000, Proteintech), light chain 3 (LC3, 1:1000, Proteintech), BNIP3 (1:1000, ZEN BIO), HIF-1α (1:1000, ZEN BIO), cyclin D1 (1:1000, ZEN BIO), PCNA (1:1000, ZEN BIO). The gel images were obtained with ECL reagent (Biosharp, China), and the results were normalized to β-actin. The intensity of bands was quantified with AlphaView SA software.

### qRT-PCR analysis

qRT-PCR analysis was performed according to our previous study. Primers for polymerase chain reaction are as follows: BNIP3, forward, TCCAGCCTCGGTTTCTATTT and reverse, AGCTCTTGGAGCTACTCCGT, GAPDH, forward, GCGGGAAATCGTGCGTGAC and reverse, CGTCATACTCCTGCTTGCTG. The mRNA level of each independently prepared RNA was determined by qRT-PCR in triplicate and normalized to GAPDH expression level. SYBR Green (Bimake, America) was used to quantify gene expression. The mRNA level of each independently prepared RNA was determined by qRT-PCR in triplicate and normalized to GAPDH level. Relative mRNA levels were expressed by the values of 2^−ΔΔCt^ as described previously (Deng et al. 2018).

### BNIP3 siRNA

The low expression of BNIP3 was achieved by invisible RNA system technology (Hanbio). The siRNA sequence of the control virus vector is as follows: The siRNA sequence of the Ctr is TTCTCCGAACGTGTCACGTAA. The siRNA sequence of the BNIP3 is GGAATTAAGTCTCCGATTA. The transfection process was carried out according to the manufacturer’s instructions. RA-FLSs were infected with virus with MOI value of 30 and cultured for 24 h. The culture medium containing virus was absorbed and replaced with fresh culture medium for 24 h. After transfection, the cells were exposed to normoxia or hypoxia for 24 h. Cell proliferation, apoptosis and autophagy were analyzed by qRT-PCR and Western blot.

### Statistical analysis

**Fig. 1:**
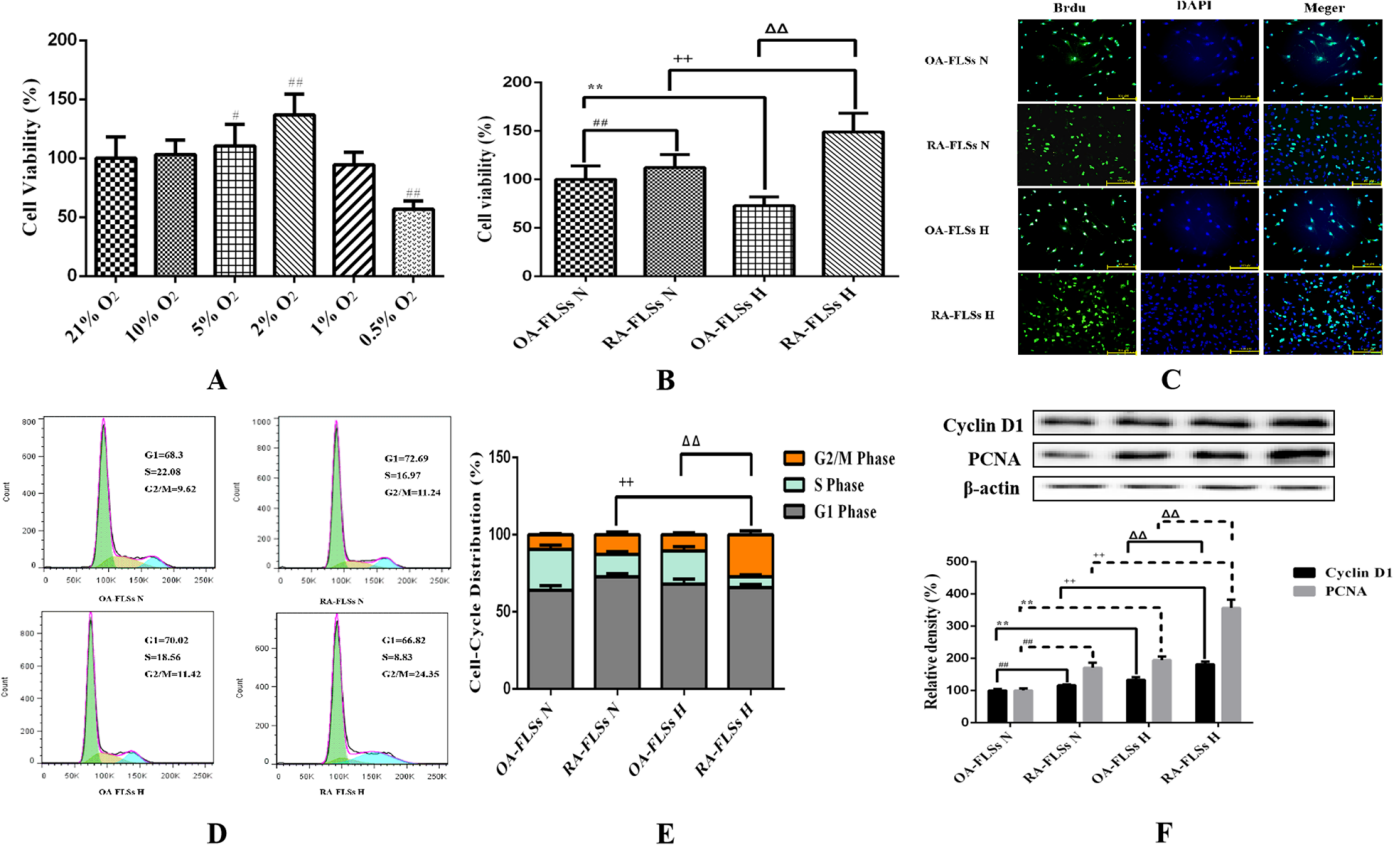
Effects of hypoxia on the proliferation of OA-FLSs or RA-FLSs. **A** The effects of different oxygen concentrations on the viability of RA-FLSs were detected by CCK-8 method (mean ± SD, n = 6 from three independent experiments); **B** The effects of hypoxia on the viability of OA-FLSs or RA-FLSs were detected by CCK-8 method (mean ± SD, n = 6 from three independent experiments); **C** BrdU was incorporated into OA-FLSs or RA-FLSs, cells were exposed to 21% and 2% O_2_ for 24 h, and then BrdU (green) and DAPI (blue) immunostaining were detected; **D** Flowcytometry analysized the cell proliferation with PI staining after cells were treated with different oxygen concentrations for 24 h; **E** Histogram depicts quantitative data from the flowcytometry analysis (mean ± SD, n = 5 from three independent experiments); **F** After exposure to different oxygen concentrations for 24 h, the expression of cyclin D1 and PCNA was analyzed by Western blot (mean ± SD; n = 6 from six independent experiments). ^#^P < 0.05, ^##^P < 0.01 represents the comparison between OA-FLSs N and RA-FLSs N; *P < 0.05, **P < 0.01 represents the comparison between OA-FLSs N and OA-FLSs H, ^+^P < 0.05, ^++^P < 0.01 represents the comparison between RA-FLSs N and RA-FLSs H, ^Δ^P < 0.05, ^ΔΔ^P < 0.01 represents the comparison between OA-FLSs H and RA-FLSs H

Graphpad prism 6.0 for windows (San Diego, California, USA) was used for statistical analysis. The statistical method used in Fig. 1A data was Student’s *t*-test, and the statistical method used for the index difference between OA/RA-FLSs before and after hypoxia was two-way ANOVA, and Bonferroni correction is used for post test with SPSS 23.0. The value of *P* < 0.05 was considered statistically significant.

## Results

### Hypoxia had different effects on the proliferation of OA-FLSs and RA-FLSs

There are significant differences in the redox state and survival state of cells under hypoxia and normoxia. In addition to apoptosis affecting the ultimate survival of cells, the effect of hypoxia on cell proliferation also affects the ultimate survival ability of cells. Therefore, we investigated the different effects of hypoxia on the proliferation of RA-FLSs and OA-FLSs. It was found that the viability of RA-FLSs increased with the decrease of oxygen concentration. When the oxygen concentration was lower than 2%, the proliferation viability decreased. Therefore, we selected 2% O_2_ as the hypoxic condition for subsequent experiments (Fig. 1A). We compared the effects of hypoxia on the survival of OA-FLSs and RA-FLSs through CCK-8 assay. After continuous exposure to hypoxia for 24 h, the cell proliferation of OA-FLSs decreased significantly, whereas the proliferation of RA-FLSs was not affected, and its cell viability increased significantly (Fig. 1B). Hypoxia relatively affected the proliferative activity of OA-FLSs, but RA-FLSs were fully tolerant to hypoxia. The proliferation of OA-FLSs and RA-FLSs were observed by BrdU incorporation method. The survival rate of RA-FLSs increased significantly after hypoxia, while the positive rate of BrdU in OA-FLSs did not change significantly (Fig. 1C). The results of flow cytometry were consistent with those of the BrdU assay (Fig. 1D, E). After hypoxia, the cell proliferation of OA-FLSs had no significant change compared with that of under normoxia (9.58% ± 0.77% vs 12.79% ± 1.67%), while the proliferation of RA-FLSs further increased, and its G2/M increased (27.15% ± 2.49% vs 10.55% ± 1.11%). We also measured the expression of cyclin D1 and proliferating cell nuclear antigen (PCNA). Cyclin D1 is G1/s-specific cyclin-D1 that is required for the progression of cell cycle from G1 to S phase. PCNA is not significantly expressed in G0–G1 phase. In late G1 phase, its expression increased significantly, peaked in S phase and decreased significantly in G2-M phase. The expressions of cyclin D1 and PCNA were significantly increased in hypoxic RA-FLSs and slightly increased in OA-FLSs (Fig. 1F). The results confirmed that although hypoxia had a relative effect on the proliferation of OA-FLSs, RA-FLSs had complete resistance.

### RA-FLSs were more resistant to hypoxia-induced apoptosis than OA-FLSs

**Fig. 2:**
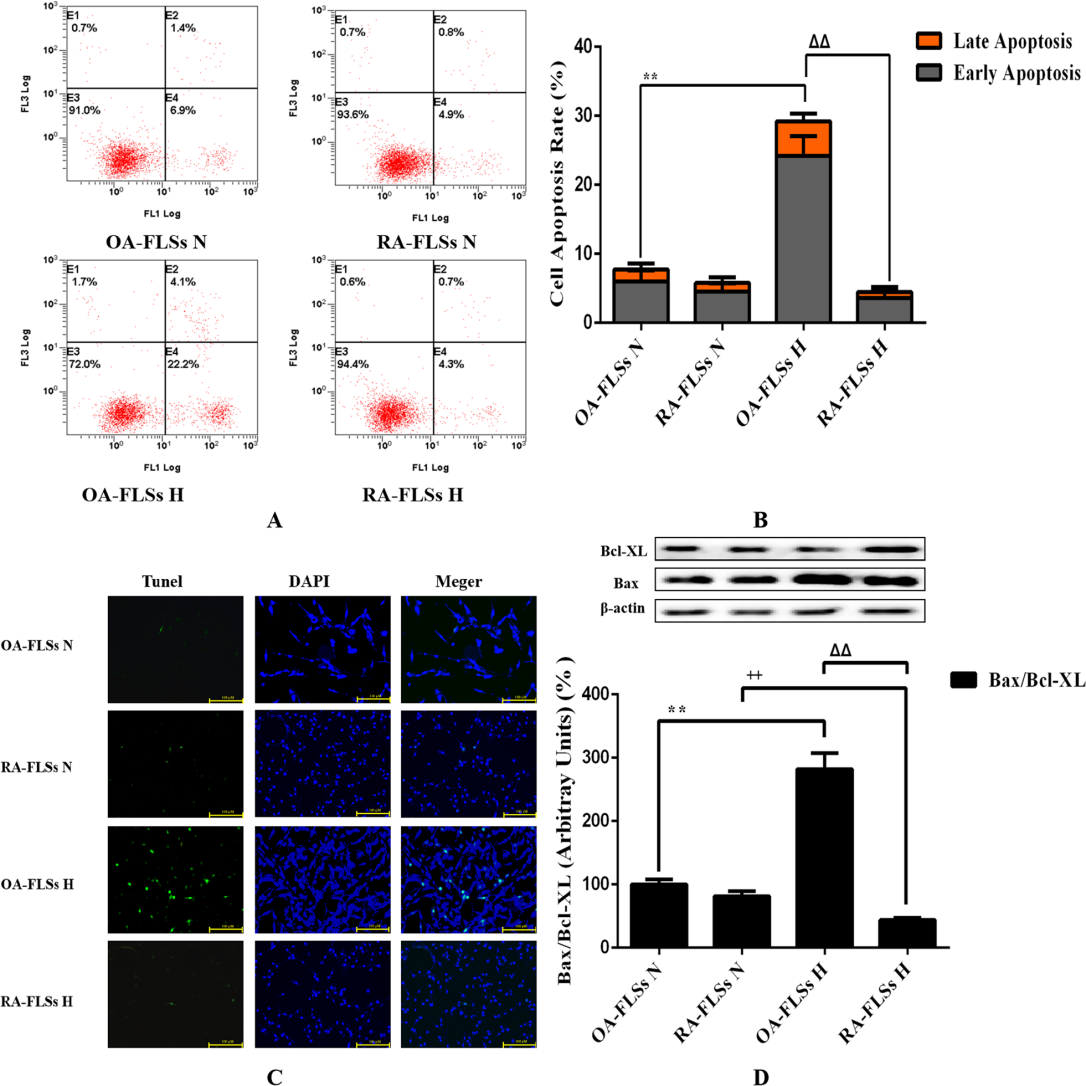
Effects of hypoxia on the apoptosis of OA-FLSs or RA-FLSs. **A** After exposed to different oxygen concentrations for 24 h, cells were analyzed by flowcytometry using AV/PI double-staining; **B** Histogram depicts the quantitative data from the AV/PI double-staining (mean ± SD, n = 6 from three independent experiments); **C** Representative fluorescence images of cells stained with TUNEL and PI to visualize apoptosis cells (Green) and nucleus (Blue), respectively; **D** The expression of Bax and Bcl-XL was analyzed by Western blot (mean ± SD, n = 6 from three independent experiments). ^#^P < 0.05, ^##^P < 0.01 represents the comparison between OA-FLSs N and RA-FLSs N; *P < 0.05, **P < 0.01 represents the comparison between OA-FLSs N and OA-FLSs H, ^+^P < 0.05, ^++^P < 0.01 represents the comparison between RA-FLSs N and RA-FLSs H, ^Δ^P < 0.05, ^ΔΔ^P < 0.01 represents the comparison between OA-FLSs H and RA-FLSs H.

Since hypoxia had different effects on the proliferative activity of OA-FLSs and RA-FLSs, we decided to evaluate the relevant mechanisms. Cell death was analyzed by different methods to determine whether it was related to apoptosis. The effects of different oxygen concentrations on the apoptosis of OA-FLSs and RA-FLSs were further observed by AV/PI double staining experiment. RA-FLSs had a low apoptosis rate under normoxia and hypoxia (7.74% ± 1.33% vs 5.78 ± 0.54%), while hypoxia significantly induced the increase of apoptosis rate of OA-FLSs (29.22% ± 1.88 vs 5.48% ± 0.71%) (Fig. 2A, B). The changes of apoptosis were further detected by TUNEL double staining. Hypoxia significantly induced apoptosis of OA-FLSs, and the rate of TUNEL positive cells increased significantly, but the rate of TUNEL positive cells in RA-FLSs was not affected by hypoxia (Fig. 2C). Since the ratio of Bax to Bcl-XL expression is an indicator of apoptosis, we calculated it at the protein level by Western blot analysis. After 24 h of hypoxia, the Bax/Bcl-XL ratio of protein level in OA-FLSs increased, while the Bax/Bcl-XL ratio in RA-FLSs decreased significantly (Fig. 2D). This indicated that OA-FLSs was more prone to apoptosis than RA-FLSs under hypoxia. Combined with the results of cell survival, it was found that although the expression of proliferation related proteins CyclinD1 and PCNA in OA-FLSs increased, the cell viability of OA-FLSs was relatively inhibited due to the significant increase of apoptosis rate.

### RA-FLSs was more prone to hypoxia-induced mitophagy than OA-FLSs

**Fig. 3:**
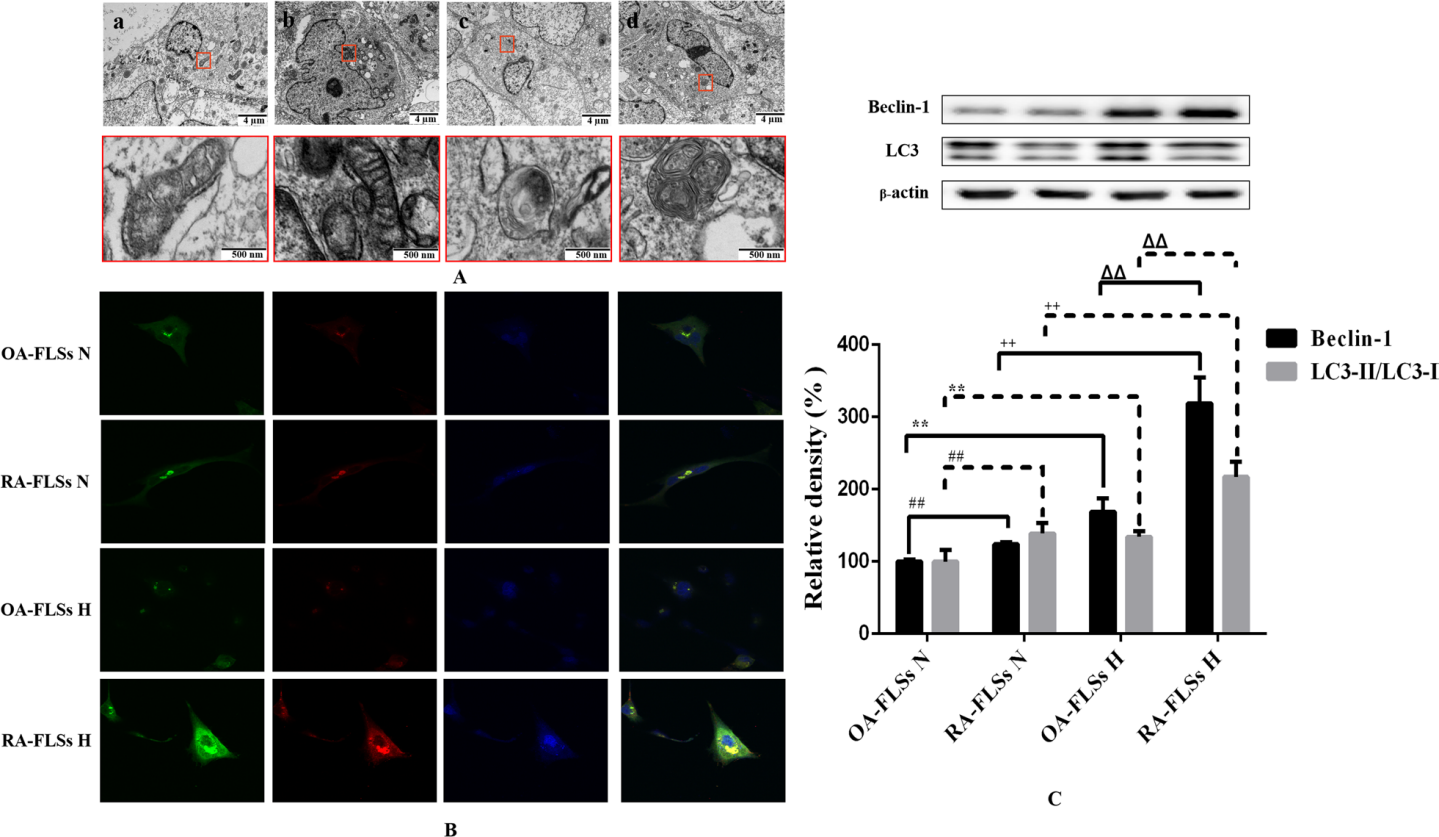
Effects of hypoxia on the mitophagy of OA-FLSs or RA-FLSs. **A** After exposed to 21%, 2% O_2_ for 24 h, cells were analysed by electron microscopy. **B** Cells were infected with EGFP-LC3 (Green) for 24 h and then exposed to 21%, 2% O_2_ for another 24 h, after that, mitochondria were marked with Mitotracker (Red), Nuclei were stained with DAPI (blue). Representative images were shown. **C** The expression of Beclin-1 and LC3 was analyzed by Western blot (mean ± SD, n = 6 from three independent experiments). ^#^P < 0.05, ^##^P < 0.01 represents the comparison between OA-FLSs N and RA-FLSs N; *P < 0.05, **P < 0.01 represents the comparison between OA-FLSs N and OA-FLSs H, ^+^P < 0.05, ^++^P < 0.01 represents the comparison between RA-FLSs N and RA-FLSs H, ^Δ^P < 0.05, ^ΔΔ^P < 0.01 represents the comparison between OA-FLSs H and RA-FLSs H

Direct observation of subcellular structural changes by electron microscope is the gold standard for the detection of mitophagy. Under normoxia, the nuclear membranes of OA-FLSs and RA-FLSs were relatively smooth, the cytoplasmic hollow vesicular structure was less, and there were a large number of mitochondria with clear double-layer membrane structure, which were spindle or oval, rich in contents and clear cristae structure. Under hypoxia, the nuclear membrane of RA-FLSs was smooth and complete, and a large number of mitochondria were oval and had normal structure and morphology, but the vacuolar structure increased, and many vacuolar structures can be observed or contacted with mitochondria. The phenomenon of enveloping mitochondria, in which vacuoled with a double membrane structure were observed to envelop darker mitochondria, that is, mitophagy. The nuclear membrane of OA-FLSs was irregularly curved, and vacuolated structures can be seen everywhere. Mitochondria with a double-layer membrane structure can be observed, but the inclusions and cristae structure almost disappeared, which indicated that the mitochondria were severely damaged and their normal structures were destroyed (Fig. 3A). LC3 is the most specific and commonly used marker molecule for autophagosomes reported today. EGFP-LC3 labeled autophagy and mitotracker labeled mitochondria were used to locate autophagosomes and mitochondria respectively, and their co-localization was used to determine the occurrence of mitophagy, which is one of the most commonly used methods in the study of mitophagy (Ren et al. 2019; Diot et al. 2018). We infected OA-FLSs/RA-FLSs with EGFP-LC3 virus, and then treated the cells with different hypoxia. After 24 h of hypoxia treatment, we labeled them with mitotracker. The results of fluorescence co-localization (Fig. 3B) showed that under hypoxia, EGFP-LC3 appeared obvious punctate and granular in RA-FLSs, which was one of the typical characteristics of autophagy induction, and the morphology of mitotracker mitochondria showed no significant change compared with that of normoxia, but the co-localization of mitotracker labeled mitochondria and EGFP-LC3 labeled autophagosomes increased, It indicated that hypoxia induced the occurrence of mitophagy. OA-FLSs showed irregular morphology, and the cell morphology changed from spindle to a large number of vacuolar structures. Mitotracker stained the mitochondria and found that the mitochondria were unevenly distributed, showing a different aggregation morphology from RA-FLSs. After treatment with different oxygen concentrations, Western Blot test results showed that compared with normoxia, the levels of LC3-II/LC3-I and Beclin-1 in RA-FLSs and OA-FLSs induced by hypoxia increased, while compared with RA-FLSs, LC3-II/LC3-I and Beclin-1 increased in OA-FLSs at a lower level (Fig. 3C). The above results indicated that hypoxia induced the occurrence of mitophagy in RA-FLSs, while mitophagy alleviated the apoptosis caused by hypoxia. Although the level of mitophagy in OA-FLSs increased, it was still not enough to eliminate the occurrence of hypoxia-induced apoptosis, which may be the reason for the significant difference in the survival rate of OA-FLSs and RA-FLSs under hypoxia.

### Hypoxia-induced responses were more pronounced in OA-FLSs than in RA-FLSs

**Fig. 4:**
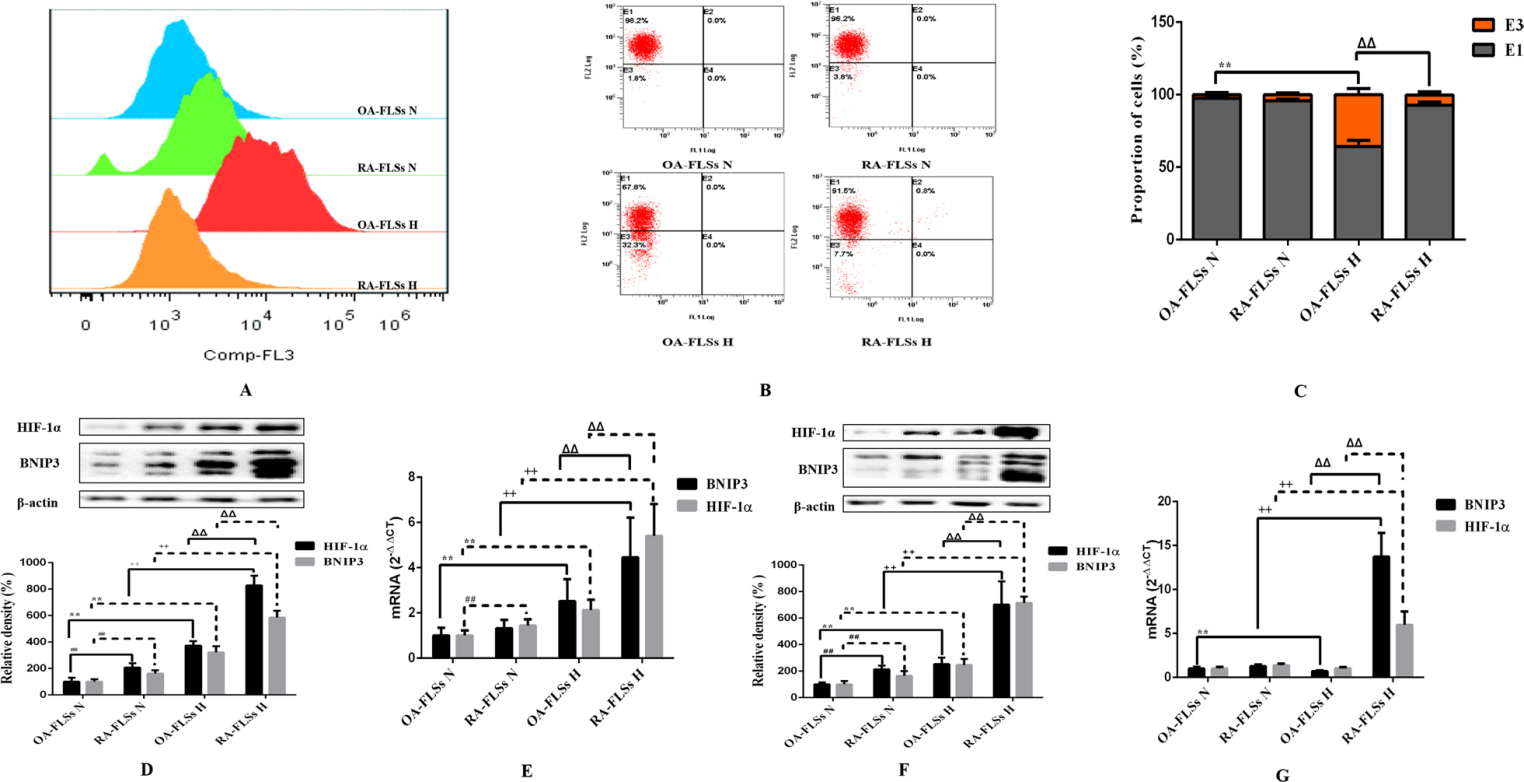
Effect of hypoxia on oxidation indexes in OA-FLSs and RA-FLSs. **A** Cells were exposed to 21% or 2% O_2_ for 24 h and assessed the ROS content by flowcytometry using 10 μM CM-H2DCFDA as a ROS probe; **B** JC-1 was used to detect the effect of hypoxia on MMP of OA-FLSs and RA-FLSs; **C** Histogram depicts the quantitative data from the JC-1 staining (mean ± SD, n = 6 from three independent experiments); **D** The expressions of HIF-1α and BNIP3 in OA-FLSs and RA-FLSs after hypoxia for 12 h were analyzed by Western blot (mean ± SD, n = 6 from three independent experiments); **E** The expressions of HIF-1α and BNIP3 in OA-FLSs and RA-FLSs after hypoxia for 12 h were analyzed by RT-qPCR (mean ± SD; n = 6 from three independent experiments); **F** The expressions of HIF-1α and BNIP3 in OA-FLSs and RA-FLSs after hypoxia for 24 h were analyzed by Western blot (mean ± SD; n = 6 from three independent experiments); **G** The expressions of HIF-1α and BNIP3 in OA-FLSs and RA-FLSs after hypoxia for 24 h were analyzed by RT-qPCR (mean ± SD; n = 6 from three independent experiments). ^#^P < 0.05, ^##^P < 0.01 represents the comparison between OA-FLSs N and RA-FLSs N; *P < 0.05, **P < 0.01 represents the comparison between OA-FLSs N and OA-FLSs H, ^+^P < 0.05, ^++^P < 0.01 represents the comparison between RA-FLSs N and RA-FLSs H, ^Δ^P < 0.05, ^ΔΔ^P < 0.01 represents the comparison between OA-FLSs H and RA-FLSs H

Hypoxia seems to have different effects on the proliferation, apoptosis and mitophagy of OA-FLSs and RA-FLSs. To determine whether these observations were related to different adaptive responses to hypoxia, we evaluated the characteristics of hypoxia in OA-FLSs and RA-FLSs. After normoxia and hypoxia treatment, flow cytometry was used to analyze the fluorescence intensity of cells stained by ROS probe molecules. The results showed that after hypoxia treatment, the fluorescence intensity of OA-FLSs stained with ROS probe molecules increased significantly, while the fluorescence intensity of RA-FLSs stained with probe molecules did not change significantly (Fig. 4A). We used the fluorescent probe JC-1 to explore the changes of MMP under different oxygen concentrations. The experimental results showed that there was no significant change in MMP in RA-FLSs after hypoxia compared with normoxia, while the MMP decreased significantly in OA-FLSs (Fig. 4B, C). In conclusion, under hypoxia, the content of ROS in RA-FLSs did not increase significantly, the MMP remained stable, and the redox balance of cells remained stable. The content of ROS in OA-FLSs increased significantly and the MMP decreased, resulting in the disorder of redox balance.

Among the many genes that are uniquely regulated at the transcriptional level of HIF-1α, BNIP3 has been implicated in the survival program of multiple cell types in response to hypoxic injury. To further analyze the redox state of cells under hypoxia, we observed the differences in the mRNA and protein expression levels of BNIP3 and HIF-1α in the two cell types after hypoxia treatment for 12 h and 24 h. As expected, the mRNA level of BNIP3 was always high in both cell types after 12 h of hypoxia. In OA-FLSs, hypoxia caused a modest increase in BNIP3 expression, which was enhanced at 12 h and then decreased at 24 h. In contrast, in RA-FLSs, we observed a dramatic increase in BNIP3 mRNA expression starting at 12 h and further increasing at 24 h (Fig. 4E, G). The Western Blot results were consistent with those of RT-qRCR. After 12 h of hypoxia, HIF-1α and BNIP3 accumulated in OA-FLSs and RA-FLSs, but at 24 h, HIF-1α and BNIP3 did not accumulate in OA-FLSs compared with normoxia, but in RA-FLSs, HIF-1α and BNIP3 continued to accumulate (Fig. 4D, F). The expression of BNIP3 protein may not only be affected by HIF-1α transcriptional regulation. These results clearly indicated that RA-FLSs were easier to adapt to hypoxia than OA-FLSs, and the regulation of BNIP3 expression may play a role in the adaptive response of cells.

### Inhibition of BNIP3 reduced RA-FLSs survival under hypoxia

**Fig. 5:**
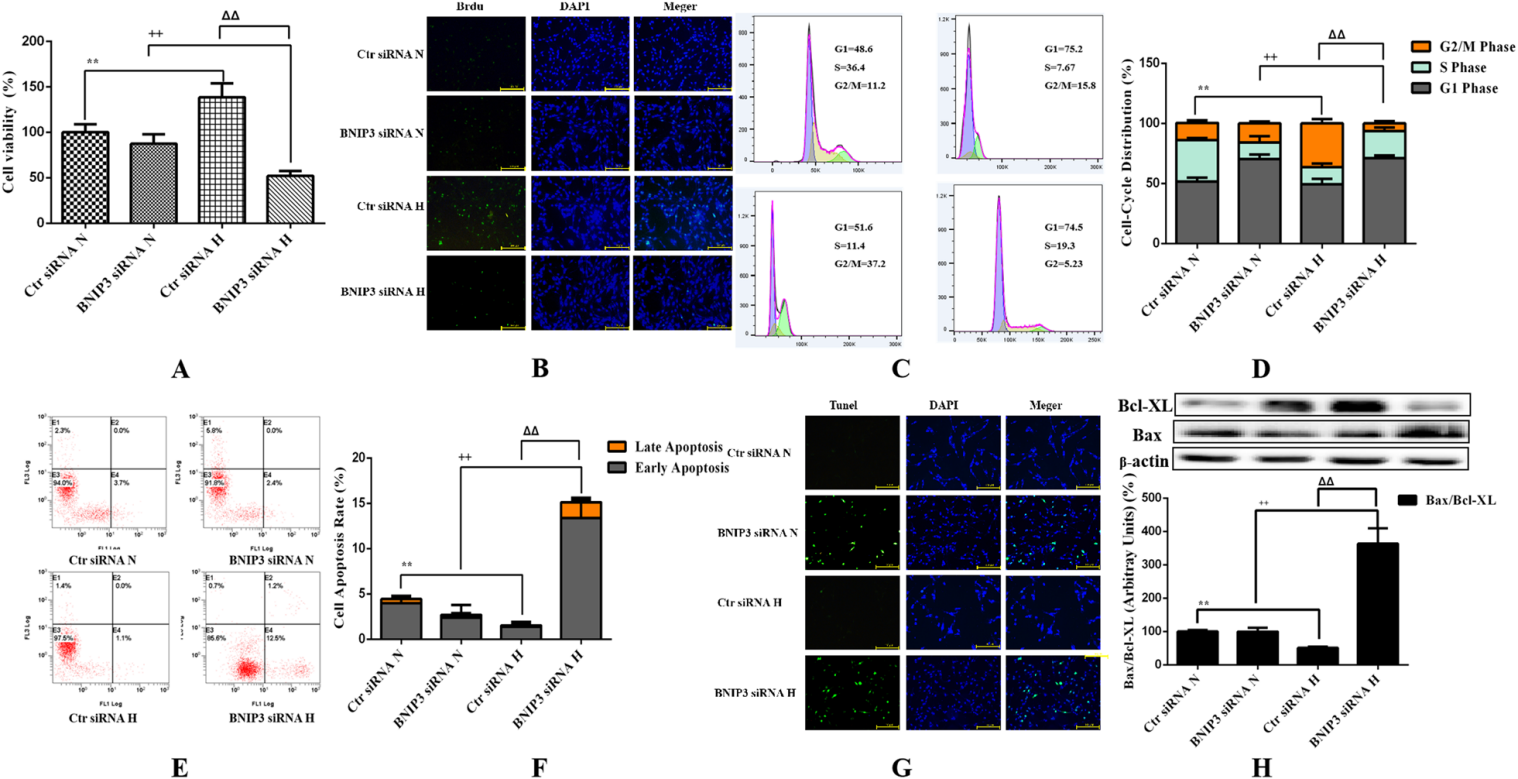
Effects of low expression of BNIP3 on proliferation and apoptosis of RA-FLSs. **A** The effects of low expression of BNIP3 on the viability of RA-FLSs were detected by CCK-8 method (mean ± SD, n = 6 from three independent experiments); **B** BrdU was incorporated into RA-FLSs, cells were exposed to 21% and 2% O_2_ for 24 h, and then BrdU (green) and DAPI (blue) immunostaining were detected; **C** Flowcytometry analysized the cell proliferation with PI staining after cells were treated with different oxygen concentrations for 24 h; **D** Histogram depicts quantitative data from the flow cytometric analysis (mean ± SD, n = 5 from three independent experiments); **E** RA-FLSs were analyzed by FCM using Annexin V/PI double-staining; **F** Histogram depicts the quantitative data from the AV/PI double-staining (mean ± SD, n = 6 from three independent experiments); **G** Representative fluorescence images of cells stained with TUNEL and Propidium Iodide to visualize apoptosis cells (Green) and nucleus (Blue); **H** The expressions of Bax and Bcl-XL was analyzed by Western blot (mean ± SD, n = 6 from three independent experiments). ^#^P < 0.05, ^##^P < 0.01 represents the comparison between Ctr siRNA N and BNIP3 siRNA N; *P < 0.05, **P < 0.01 represents the comparison between Ctr siRNA N and Ctr siRNA H, ^+^P < 0.05, ^++^P < 0.01 represents the comparison between BNIP3 siRNA N and BNIP3 siRNA H, ^Δ^P < 0.05, ^ΔΔ^P < 0.01 represents the comparison between Ctr siRNA H and BNIP3 siRNA H

**Fig. 6:**
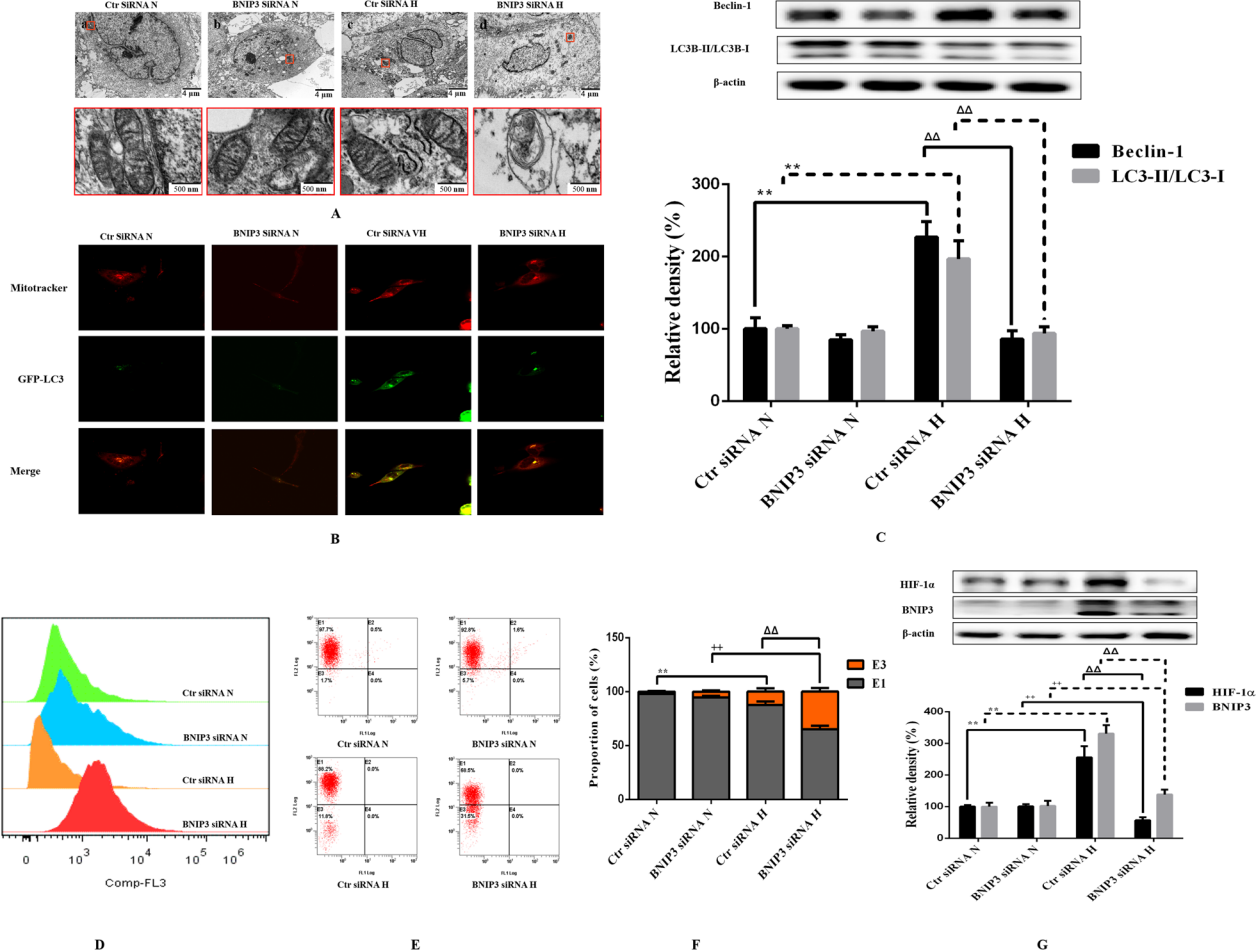
Effects of low expression of BNIP3 on the mitophagy and oxidation indexes of RA-FLSs. **A** RA-FLSs were analysed by electron microscopy. **B** RA-FLSs were infected with EGFP-LC3 (Green) for 24 h and then exposed to 21% and 2% O_2_ for another 24 h, after that, mitochondria were marked with Mitotracker (Red). Representative images are shown. **C** The expression of Beclin-1 and LC3 was analyzed by Western blot (mean ± SD, n = 6 from three independent experiments); **D** RA-FLSs were exposed to 21% O_2_ or 2% O_2_ for 24 h and assessed the ROS content by FCM using 10 μM CM-H2DCFDA as a ROS probe; **E**C-1 was used to detect the effect of hypoxia on MMP of RA-FLSs; **F** Histogram depicts the quantitative data from the JC-1 staining (mean ± SD, n = 6 from three independent experiments); **G** The expressions of HIF-1α and BNIP3 in RA-FLSs after hypoxia for 24 h were analyzed by Western blot (mean ± SD, n = 6 from three independent experiments). ^#^P < 0.05, ^##^P < 0.01 represents the comparison between Ctr siRNA N and BNIP3 siRNA N; *P < 0.05, **P < 0.01 represents the comparison between Ctr siRNA N and Ctr siRNA H, ^+^P < 0.05, ^++^P < 0.01 represents the comparison between BNIP3 siRNA N and BNIP3 siRNA H, ^Δ^P < 0.05, ^ΔΔ^P < 0.01 represents the comparison between Ctr siRNA H and BNIP3 siRNA H

In order to better determine the role of BNIP3 in the survival of RA-FLSs under hypoxia, we suppressed the expression of BNIP3 by siRNA technology to observe its effects on the proliferation, apoptosis and mitophagy of RA-FLSs. Under normoxia and hypoxia, the inhibition of BNIP3 resulted in a significant decrease in the expression of BNIP3, while hypoxia still significantly induced the expression of BNIP3 in the Ctr siRNA group. Inhibition of BNIP3 expression was associated with decreased cell proliferation (Fig. 5A–D) and enhanced apoptosis (Fig. 5E–H), and was closely related to the reduction of mitophagy caused by the inhibition of BNIP3 in RA-FLSs under hypoxia (Fig. 6A–C). In fact, the disorder of cell redox balance in the hypoxic RA-FLSs silenced by BNIP3 was accompanied by the accumulation of ROS and the decrease of MMP, while the redox balance of RA-FLSs in the hypoxic Ctr siRNA group remained stable (Fig. 6D–G). Our results again indicated that BNIP3 played a key role in the survival of RA-FLSs with pathological and hyperproliferative properties.

## Discussion

Studies confirmed that RA-FLSs have special hypoxia tolerance, and RA-FLSs show abnormal proliferation activity in the joint hypoxic microenvironment of RA (Yu et al. 2019). Our study showed that RA-FLSs were functionally different from OA-FLSs, which was related to the abnormal hyperplasia of joint synovium in the progression of RA. We compared the adaptive response of FLSs from OA patients and RA patients to hypoxia. This is the first report that RA-FLSs were more resistant to hypoxia than OA-FLSs in terms of cell viability (Fig. 1B–F), and were more prone to mitophagy (Fig. 3A–C) and less prone to apoptosis (Fig. 2A–D). In our study, we determined that OA-FLSs were less resistant to cell death than RA-FLSs due to long-term exposure to hypoxia. Actually, short-term exposure to hypoxia did not induce cell death of OA-FLSs or RA-FLSs, that is, OA-FLSs resisted hypoxia induced cell death after 12 h of exposure. However, after 24 h, the survival rate of OA-FLSs decreased, but RA-FLSs continued to resist hypoxia. RA-FLSs expressed higher levels of Cyclin D1 and PCNA compared with OA-FLSs (Fig. 1F). Cyclin D1 is G1/s-specific Cyclin-D1, which is necessary for the progression of cell cycle from G1 to S phase. PCNA is closely related to cell DNA synthesis and plays an important role in the initiation of cell proliferation, and is a good indicator to reflect the state of cell proliferation (Converse and Thomas 2021; Tao et al. 2021). The results showed that the increase expression of Cyclin D1 and PCNA in RA-FLSs were closely associated with increased cell proliferation. Regarding the detection of apoptosis, we observed that changes in the ratio of Bax/Bcl-xL was negatively correlated with cell survival (Fig. 2D). Our results indicated that hypoxia-induced death of OA-FLSs may be attributed to apoptosis, which was consistent with the hypothesis that RA-FLSs were more resistant to hypoxia-induced cell death. It was important to highlight the marked increase in Beclin-1 expression and LC3B-II/LC3B-I ratio in hypoxic RA-FLSs (Fig. 3C). As mentioned earlier, mitophagy may be considered as a pro-survival adaptive response in several cell types. This may further explain why OA-FLSs is less resistant to hypoxia-induced cell death than RA-FLSs, which are more prone to mitophagy.

Previous studies have clearly clarified how cells adapt to hypoxia by expressing genes tightly regulated by HIF-1α, including BNIP3, GLUT-1, CAIX and MCT-4, in order to survive in an hostile microenvironment (Filippi et al. 2018). OA-FLSs accumulated less HIF-1α than RA-FLSs, and RA-FLSs expressed higher levels of BNIP3 than OA-FLSs, which supported the hypothesis that RA-FLSs showed better metabolic and mitophagy adaptive response to hypoxia (Fig. 4D, E). In fact, BNIP3 has been described as a key hypoxia-induced molecule participated in the switch of mitophagy, promoting cell survival and avoiding apoptosis (Tang et al. 2019). Our results are completely consistent with previous reports, indicating that hypoxia-induced mitophagy is a survival process, which is associated with the expression of BNIP3. It should be emphasized that the expression of BNIP3 in RA-FLSs is higher than that of OA-FLSs even under normoxia. Our results clearly defined the overexpression of BNIP3 in RA-FLSs under hypoxia (Fig. 4D–G). When BNIP3 was low expressed in RA-FLSs by siRNA transfection, the proliferative activity of RA FLSs was decreased (Fig. 5A–D) and apoptosis was increased (Fig. 5E–H), which further confirmed the protective effect of BNIP3 on RA-FLSs under hypoxia. The present study showed that apoptosis was increased when the expression of BNIP3 was inhibited in RA-FLSs. Taken together, our results suggested that understanding the mechanism of hypoxia adaptation of different FLSs may have important therapeutic implications.

BNIP3 is an outer mitochondrial membrane protein that localizes to mitochondria upon expression. It is also an important signal molecule for the activation of hypoxia-induced mitophagy. How do they activate mitophagy? After a series of studies, it is found that Beclin-1 plays an important role in the induction and activation of BNIP3-mediated autophagy pathway (Lee et al. 2020). Beclin-1, a main inducer of autophagy, plays an important role in the formation of autophagic precursor structures and regulates the formation of autophagic precursor (Hill et al. 2019). Beclin-1 and BNIP3 are members of the Bcl-2 family, both of which are BH3-only subfamily proteins with BH3 domains. Therefore, Beclin-1 binds to anti-apoptotic proteins Bcl-2 or Bcl-XL through its BH3 domain. When hypoxia-induced BNIP3 is highly expressed, BNIP3 competes with Beclin-1 through its BH3 structure and binds to Bcl-2 or Bcl-XL. Therefore, Beclin-1 is released from the Bcl-2/Beclin-1 or Bcl-XL/Beclin-1 complex on the mitochondria, and the free Beclin-1 forms a PI3K complex with Vps34, Ambra1 and other proteins for activation. The PI3K/Akt pathway regulates the position of downstream autophagy-related ATG proteins in autophagy precursor and isolation membrane to activate mitophagy (Niu et al. 2019; Won et al. 2015). By knocking down the expression of Beclin-1 and ATG-5, the HIF-1α/BNIP3 pathway-dependent pro-cell survival can be inhibited, which proves that the PI3K/ATG pathway mediated by Beclin-1 plays an important role in the autophagy activated by HIF-1α/BNIP3 (Niu et al. 2019; Zhang et al. 2008).

The understanding of BNIP3 in the regulation of mitophagy is not only to regulate Beclin-1 through BH3 domain to activate autophagy. BNIP3 also activates mitophagy by inducing MMP loss or depolarization and directly interacting with autophagy connecting molecule LC3 (Zeng et al. 2021; Shi et al. 2014). When autophagy is activated, the cytoplasmic LC3B-I is cleaved, fatty and inserted into the autophagosome membrane as LC3-II. Therefore, the increase in the number of LC3-II proteins with small molecular weight and the increase in the ratio of LC3-II/LC3-I are markers of autophagy, which is related to the increase in the number of autophagosomes.

Some studies have also proved that the oxidative imbalance caused by the large production of ROS in mitochondria is the main reason for the induction of adaptive mitophagy (Zeb et al. 2021). Oxidative stress caused by ROS can not only induce mitophagy through HIF-1 mediated pathway, but also regulate apoptosis by activating other signal molecule controlled pathways (Forte et al. 2021; Sumi et al. 2022). At present, there are differences on the function of BNIP3 in promoting survival or apoptosis. A variety of proteins that regulate cell death in the Bcl-2 family have been found to have complex post transcriptional regulation, including phosphorylation and ubiquitination at protein level and shear modification at RNA level (Song et al. 2019; Lv et al. 2021). Its post transcriptional modification has changed the protein function, which explained why pro-apoptotic factors in a variety of Bcl-2 families were highly expressed in diseases. In the research literature of BNIP3, the 30 kDa band has always been considered as the standard band to judge BNIP3. The immunoblot results of exogenous constructed plasmid also confirmed that BNIP3 appeared at 30 kDa (Ray et al. 2000). In our experiment, in addition to 30 kDa and 60 kDa BNIP3 monomers and homodimers, multiple bands with different mobility were observed between 30 and 21.5 kDa in SDS-PAGE electrophoresis. This phenomenon can be observed in Figs. 4 and 6. The results confirmed that BNIP3 may have a regulatory pathway of post-translational modification under hypoxia. It has been reported that there may be phosphorylation modification in BNIP3, and phosphorylation modification may be the key factor affecting a variety of different mobility bands of BNIP3 in PAGE electrophoresis (Mellor et al. 2010). We speculated that post transcriptional modification is also suitable to explain the differences of BNIP3 in promoting cell survival or death. However, there are few reports on the post transcriptional modification of BNIP3. Could this band-type change in BNIP3 be linked to cell survival or mitophagy? We need to do further experimental verification in future research.

## Conclusion

**Fig. 7:**
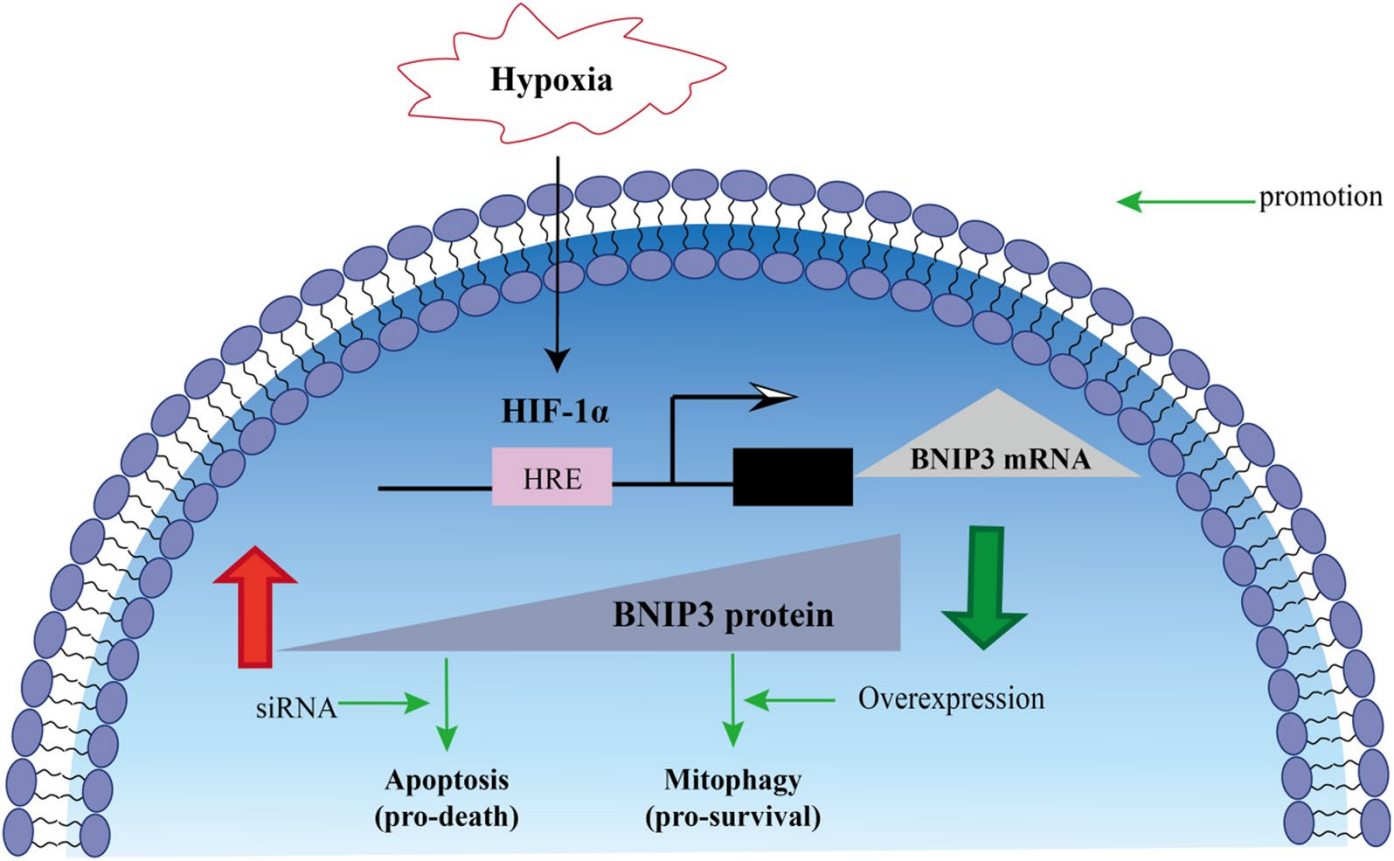
Under hypoxic conditions, the occurrence of mitophagy and the regulation of BNIP3. Under hypoxia, RA-FLSs mediated mitophagy through high expression of BNIP3 to eliminate excess ROS in cells, maintain redox balance, reduce the occurrence of apoptosis, and promote cell survival. The expression of BNIP3 in OA-FLSs was relatively small, and the mitophagy mediated by it was not enough to eliminate excess ROS in cells, resulting in increased apoptosis and decreased cell survival

In summary, this study proved that RA-FLSs cleared ROS accumulated in cells through mitophagy under hypoxia to maintain the balance of redox, reduce apoptosis and promote cell survival. While the mitophagy in OA-FLSs was not enough to remove the accumulated ROS, and the redox imbalance in OA-FLSs led to the increase of apoptosis and the decrease of cell viability. The difference of mitophagy between OA-FLSs and RA-FLSs under hypoxia was mediated by BNIP3 expression (Fig. 7).

## Data Availability

The data and materials used to support the findings of this study are available from the corresponding author upon request.

## Abbreviations

BNIP3: Bcl-2 and adenovirus E1B 19 kDa interacting protein 3
BrdU: Bromodeoxyuridine
CCK-8: Cell counting kit-8
FBS: Fetal bovine serum
FLSs: Fibroblast-like synovial cells
HIF-1: Hypoxia-inducible factor 1
h: Hour
LC3: Light chain 3
Min: Minute
MMP: Mitochondrial membrane potential
OA: Osteoarthritis
OA-FLSs: Fibroblast-like synovial cells from patients with osteoarthritis
PCNA: Proliferating cell nuclear antigen
PI: Propidium iodide
RA: Rheumatoid arthritis
RA-FLSs: Fibroblast-like synovial cells from patients with rheumatoid arthritis
ROS: Reactive oxygen species
